# Correlation of capecitabine-induced skin toxicity with treatment efficacy in patients with metastatic colorectal cancer: results from the German AIO KRK-0104 trial

**DOI:** 10.1038/bjc.2011.227

**Published:** 2011-07-12

**Authors:** S Stintzing, L Fischer von Weikersthal, U Vehling-Kaiser, M Stauch, H G Hass, H Dietzfelbinger, D Oruzio, S Klein, K Zellmann, T Decker, M Schulze, W Abenhardt, G Puchtler, H Kappauf, J Mittermüller, C Haberl, C Giessen, N Moosmann, V Heinemann

**Affiliations:** 1 Medical Department III, University of Munich, Klinikum Muenchen-Grosshadern, Marchioninistrasse 15, D-81377 Munich, Germany; 2 Gesundheitszentrum, St Marien GmbH, 92224 Amberg, Germany; 3 Onkologische Praxis, 84028 Landshut, Germany; 4 Onkologische Praxis, 96317 Kronach, Germany; 5 Marienhospital, 70199 Stuttgart, Germany; 6 Onkologische Praxis, 82211 Herrsching, Germany; 7 Klinikum, 86156 Augsburg, Germany; 8 Klinikum, 95445 Bayreuth, Germany; 9 Schlossbergklinik, 87534 Oberstaufen, Germany; 10 Onkologische Praxis, 88214 Ravensbrück, Germany; 11 Onkologische Praxis, 02763 Zittau, Germany; 12 Onkologische Praxis MPO, 80335 München, Germany; 13 Klinikum, 83022 Rosenheim, Germany; 14 Onkologische Praxis, 82319 Starnberg, Germany; 15 Onkologische Praxis, 82110 Germering, Germany; 16 Klinikum, 94315 Straubing, Germany

**Keywords:** capecitabine, colorectal cancer, cetuximab, skin toxicity

## Abstract

**Background:**

The AIO KRK-0104 randomised phase II trial investigated the efficacy and safety of two capecitabine-based regimens: combination of capecitabine and irinotecan (CAPIRI) plus cetuximab (CAPIRI-C) and combination of capecitabine with oxaliplatin (CAPOX) plus cetuximab (CAPOX-C) in the first-line treatment of metastatic colorectal cancer (mCRC). Treatment-related skin toxicity (ST) was evaluated separately for capecitabine and cetuximab. The present analysis investigates the correlation of capecitabine-attributed ST (Cape-ST) and parameters of treatment efficacy.

**Methods:**

Patients with mCRC were randomised to cetuximab (400 mg m^−2^, day 1, followed by 250 mg m^−2^ weekly) plus CAPIRI (irinotecan 200 mg m^−2^, day 1; capecitabine 800 mg m^−2^, twice daily, days 1–14, every 3 weeks), or cetuximab plus CAPOX (oxaliplatin 130 mg m^−2^, day 1; capecitabine 1000 mg m^−2^, twice daily, days 1–14, every 3 weeks).

**Results:**

Of 185 recruited patients, 149 (CAPIRI-C, *n*=78; CAPOX-C, *n*=71) received study treatment beyond the first tumour assessment and were evaluable for efficacy. Capecitabine-attributed ST, predominantly hand–foot syndrome, was observed in 32.2% of patients. Capecitabine-attributed ST grade 1–3 was associated with a significantly higher disease control rate (DCR) (97.9 *vs* 86.1%, *P*=0.038) compared with grade 0 toxicity. Moreover, Cape-ST grade 1–3 related to a markedly longer progression-free survival (PFS) (9.9 *vs* 5.6 months, *P*<0.001) and overall survival (OS) (32.8 *vs* 22.4 months, *P*=0.008). Separate analyses of treatment arms indicated that the effect of Cape-ST on PFS remained significant for both arms, whereas the effect on OS remained apparent as a strong trend.

**Conclusion:**

This analysis supports the hypothesis that for the evaluated regimens, a correlation exists between Cape-ST and treatment efficacy regarding DCR, PFS, and OS.

Fluoropyrimidines such as 5-fluorouracil (5-FU) or capecitabine are effective and well-established agents in the treatment of metastatic colorectal cancer (mCRC). Capecitabine is an oral prodrug that is metabolised to the active 5-FU in a three-step enzymatic conversion (Miwa *et al*, 1998). The last and rate-limiting step of drug activation is catalysed intracellularly by thymidine phosphorylase (TP) (Diasio, 2002). Tumour cells notably express higher levels of TP than normal tissue, which may be responsible for some degree of selective anti-tumour activity (Schuller *et al*, 2000).

Several phase III trials have demonstrated that the combination of capecitabine with oxaliplatin (CAPOX) has similar activity as the combination of oxaliplatin with an infusional 5-FU regimen (FOLFOX) (Diaz-Rubio *et al*, 2007; Porschen *et al*, 2007; Cassidy *et al*, 2008). The combination of capecitabine and irinotecan (CAPIRI) has never been formally compared with an infusional 5-FU-containing regimen. However, the efficacy and safety of CAPIRI are documented by a number of independent clinical studies (Bajetta *et al*, 2004; Rea *et al*, 2005; Koopman *et al*, 2007; Moosmann *et al*, 2011).

Capecitabine notably induces ST, specifically hand–foot syndrome (HFS) and nail changes in a dose-dependent manner. Although HFS is not a life-threatening toxicity, it may induce a substantial decrease of quality of life and, accordingly, may cause a delay or discontinuation of treatment (Heo *et al*, 2004). According to data from phase II and phase III trials, HFS grade 1–3 occurs at a rate of 43–71%, whereas grade 3 HFS has an incidence of 5–24% (Wolf *et al*, 2010). Up to now, only little information is available relating capecitabine-induced ST (Cape-ST) to treatment efficacy. The present analysis investigates this topic in the AIO KRK-0104 study. This randomised phase II trial investigated two capecitabine-based regimens: CAPIRI plus cetuximab (CAPIRI-C) and CAPOX plus cetuximab (CAPOX-C) in the first-line treatment of mCRC (Moosmann *et al*, 2011). As it was known that ST was an important predictor of treatment efficacy in cetuximab-treated patients, it was carefully and prospectively evaluated within the study protocol. On the basis of this premise it was possible to perform a separate evaluation of ST induced by capecitabine or cetuximab according to the judgment of the treating physician.

## Materials and methods

This explorative analysis evaluated patients of the randomised AIO KRK-0104 phase II study. A total of 185 patients from 35 German centres were included. In both the study arms, cetuximab was given at an initial dose of 400 mg m^−2^ as a 120-min infusion, followed by weekly infusions of 250 mg m^−2^ over 60 min. Patients in the CAPIRI-C arm received chemotherapy with CAPIRI, that is, oral capecitabine 800 mg m^−2^, twice daily on days 1–14, followed by a 1-week rest period plus irinotecan 200 mg m^−2^ as a 30-min intravenous infusion on day 1. In patients aged >65 years, doses were further reduced by 20%. Patients in the CAPOX-C arm received capecitabine 1000 mg m^−2^, twice daily on days 1–14, followed by a 1-week rest period plus oxaliplatin 130 mg m^−2^ as a 120-min intravenous infusion on day 1. Treatment cycles were repeated every 3 weeks until disease progression or unacceptable toxicity. Response was evaluated with consistent imaging techniques (MRI or CT scan) every 6 weeks (two cycles) according to the RECIST criterions. Only patients that were on study for more than 6 weeks and therefore had at least one tumour assessment were included into this analysis. These patients were defined as assessable for treatment efficacy.

### Evaluation of skin toxicity

Skin toxicity was evaluated using the NCI-CTCAE (National Cancer Institute Common Toxicity Criteria of Adverse Events) version 3.0 at any treatment cycle. Possible items were: desquamation/rash, nail changes, HFS, and ‘other skin toxicities’. Investigators were asked to specifically relate toxicity to the study medication. As previously reported by others, Cape-ST was defined as HFS or any other reported ST when indicated as capecitabine related (Tol *et al*, 2009). By contrast, cetuximab-related ST was defined as ST, which was marked by the investigators as cetuximab-related, with the exception of HFS. We analysed the time to first occurrence and time of maximum grade ST by treatment cycle, and its predictive value with regard to treatment efficacy. Furthermore, ST was grouped into clinically nonsignificant ST (grade 0–1) and clinically relevant ST (grade 2–4), requiring treatment or discontinuation of the study medication.

To exclude the possibility that Cape-ST is just an indicator of longer treated patients, we also calculated survival (progression-free survival (PFS) and overall survival (OS)) for patients that were on treatment until the first (two cycles) and second (four cycles) tumour assessment.

### Statistics

Correlation analyses were performed regarding Cape-ST and efficacy parameters such as overall response rate (ORR), disease control rate (DCR), PFS, and OS. Differences in ORR and DCR between the different treatment groups, CAPIRI-C and CAPOX-C, were evaluated using Fisher's exact test within an exploratory analysis. Progression-free survival and OS were tested using the Kaplan–Meier method and the logrank test. SPSS PASW 18.0 (SPSS Inc., Chicago, IL, USA) software was used for statistical analyses.

## Results

### Patient characteristics

Of 185 randomised patients, 149 patients received chemotherapy until the first tumour assessment and were defined as the efficacy-assessable population. Patient characteristics were balanced between treatment arms with regard to median age, sex, Karnofsky performance status, visceral metastasis, and number of metastatic disease sites.

### Incidence of skin toxicity

Skin toxicity attributed to capecitabine by investigators was observed in 48 out of 149 (32.2%) patients. Hand–foot syndrome as the main Cape-ST was diagnosed in 46 patients (31%) and nail changes in 12 patients (8%). Accordingly, among 48 patients there were only 2 patients presenting with nail changes attributed by investigators to capecitabine who did not suffer from a HFS. Overall, no grade 4 ST was observed. Capecitabine-specific ST grade 1–3 was observed to a greater extent in the CAPOX-C compared with the CAPIRI-C in 25.6% of patients in the CAPIRI-C arm, and in 39.4% of the CAPOX-C arm (Table 1). This finding most likely relates to the higher dose of capecitabine used in the CAPOX-C arm. By contrast, cetuximab-associated ST was documented in 142 out of 149 (95.3%) of assessable patients.

### Treatment delay and dose reductions

Of 920 evaluated treatment cycles, 539 were applied in patients with grade 0 Cape-ST and 381 cycles in patients with grade 1–3 Cape-ST. Dose reductions of capecitabine were significantly more frequent in patients with Cape-ST (45.1 *vs* 29.3%, *P*<0.0001). On the contrary, delays of treatment were documented less often in Cape-ST patients (15.5 *vs* 21.0%, *P*=0.04).

### Kinetics of skin toxicity

**Figure 1:**
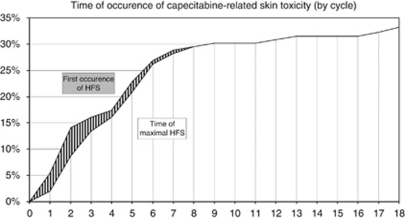
Time of occurrence of capecitabine-related skin toxicity by cycle.

Capecitabine-attributed ST was diagnosed after median treatment duration of three cycles (Figure 1). Maximal Cape-ST developed at a slower rate, and median time to maximal Cape-ST was five treatment cycles. To some extent, the kinetics of Cape-ST paralleled those of cetuximab-ST. However, cetuximab-ST developed faster and was already documented after a median time of one treatment cycle. Median time to maximal cetuximab-ST was two cycles.

### Correlation of skin toxicity and response to treatment

Tumour response to systemic chemotherapy was nearly identical when patients received CAPIRI-C (52.6, 95% CI: 40.9–64.0) or CAPOX-C (59.2, 95% CI: 46.8–70.7) (Moosmann *et al*, 2011) (Table 2). We therefore analysed both treatment arms together and compared Cape-ST in both treatment arms with parameters of efficacy (Table 2). When the subgroup of Cape-ST grade 0 (*n*=101) was compared with patients with Cape-ST grade 1–3, ORR did not significantly differ (54.5 *vs* 58.3%, *P*=0.725). However, significantly more patients achieved a DCR in the Cape-ST grade 1–3 subgroup (97.9 *vs* 86.1%, *P*=0.038).

### Correlation of skin toxicity to survival parameters

**Figure 2:**
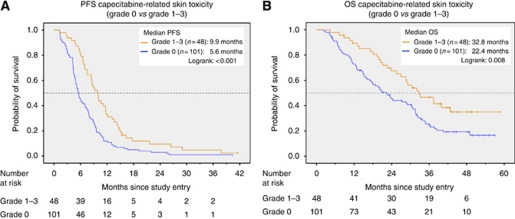
(**A**) Progression-free survival, capecitabine-related skin toxicity (grade 0 *vs* grade 1–3). (**B**) Overall survival, capecitabine-related skin toxicity (grade 0 *vs* grade 1–3).

In the combined analysis of both treatment arms, the higher the grade of Cape-ST to be observed, the longer the PFS and OS were (Table 3). Progression-free survival was significantly longer when patients with Cape-ST grade 1–3 were compared with Cape-ST grade 0 (9.9 *vs* 5.6 months, *P*<0.001; Table 3B). Similarly, also OS was markedly longer in the Cape-ST grade 1–3 group than in the Cape-ST grade 0 group (32.8 *vs* 22.4%, *P*=0.008) (Figures 2A and B; Table 3B).

Even when survival times were recalculated for patients who were on treatment until the second tumour assessment (four cycles or 6 weeks), data for PFS (6.3 *vs* 9.9 months, logrank *P*<0.001) and OS (27.3 *vs* 33.2 months, logrank *P*=0.034) remained significant.

**Figure 3:**
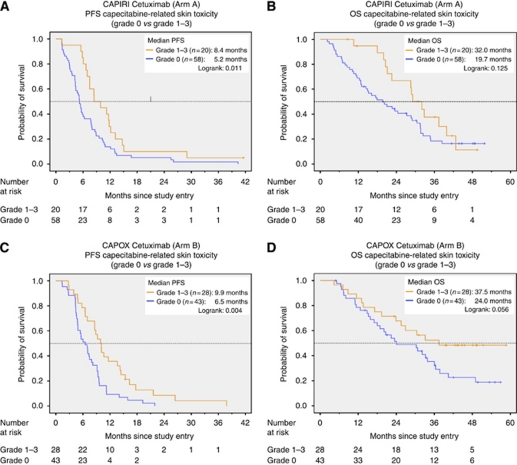
(**A**) CAPIRI cetuximab (Arm A) PFS, capecitabine-related skin toxicity (Cape-ST) (grade 0 *vs* grade 1–3). (**B**) CAPIRI cetuximab (Arm A) OS, Cape-ST (grade 0 *vs* grade 1–3). (**C**) CAPOX cetuximab (Arm B) PFS Cape-ST (grade 0 *vs* grade 1–3). (**D**) CAPOX cetuximab (Arm B) OS, Cape-ST (grade 0 *vs* grade 1–3).

This effect was independently observed also when the treatment arms were evaluated separately (Tables 3C and D). The impact of Cape-ST on PFS remained significant for CAPIRI-C (8.5 *vs* 5.2 months, *P*=0.011) and for CAPOX-C (9.9 *vs* 6.5 months, *P*=0.004) (Figures 3A and C). The effect of Cape-ST on OS also remained evident as a strong trend for CAPIRI-C (32.0 *vs* 19.7 months) and for CAPOX-C (37.5 *vs* 24.0 months) (Figures 3B and D; Tables 3C and D).

## Discussion

The present study investigated Cape-ST as an early indicator of treatment efficacy in patients receiving either CAPIRI-C or CAPOX-C. Although both, capecitabine and cetuximab cause ST, they mostly present with a different phenotype (e.g., HFS *vs* acneiform rash). However, there is also some possibility of overlap in ST induced by these agents. Within this analysis, it was therefore decided to follow the investigators’ judgement. Capecitabine-related ST was, accordingly, defined as HFS or any other reported ST when an association with capecitabine was stated by the treating physician.

### Incidence of capecitabine-induced skin toxicity

Capecitabine-induced ST was documented in 32% (48 out of 149) of assessable patients. When both groups were evaluated together, grade 3 Cape-ST was observed in 6.7% (10 out of 149) of patients. In the CAPOX-C arm, grade 3 Cape-ST occurred at a rate of 11.3%, which is in keeping with previous reports. According to Cassidy *et al* (2008), chemotherapy with CAPOX alone induced grade 3 HFS at a rate of 6%. Borner *et al* (2008) reported a randomised phase II trial comparing CAPOX with CAPOX-C. The rate of grade 3–4 HFS was 5% in patients receiving CAPOX and 3% when cetuximab was added. Owing to a higher dose of capecitabine, the rate of grade 3–4 HFS was higher in the CAIRO2 study, but in this study the addition of cetuximab did not increase its incidence (18 *vs* 16%) (Tol *et al*, 2008).

Certainly, the question remains to what extent capecitabine and cetuximab interact at the level of ST. An evaluation of the published literature leads to the following conclusions: (1) There is no indication that cetuximab applied as a single agent induces HFS (Cunningham *et al*, 2004; Jonker *et al*, 2007). (2) There is no evidence from comparative studies to suggest that cetuximab enhances the capecitabine-induced HFS (Borner *et al*, 2008; Tol *et al*, 2009).

### Dose dependence of hand–foot syndrome

Capecitabine-induced ST occurs as a dose-dependent event (Lassere and Hoff, 2004; Gressett *et al*, 2006). As a higher dose of capecitabine was administered in the CAPOX-C-compared with the CAPIRI-C arm (1000 mg m^−2^ b.i.d. *vs* 800 mg m^−2^ b.i.d.), Cape-ST was more frequently observed in the CAPOX-C arm (39.4 *vs* 25.6%, *P*=0.81).

Although it can be argued that the poor response of patients without Cape-ST may just be a result of underdosing, it needs to be pointed out that all patients were treated according to the study protocol. Therefore, none of those were underdosed ‘per protocol’ at the start of the trial. When treatment patterns were analysed, it became apparent that patients with any Cape-ST had a significantly higher percentage of cycles with dose reductions of capecitabine (45.1 *vs* 29.3%, *P*<0.0001). This leads to the conclusion that the average capecitabine dose per cycle was even lower in patients with Cape-ST than in those without any Cape-ST. Possibly as a result of dose reductions, treatment delays occurred less frequently in patients with Cape-ST. As baseline characteristics of both patient groups did not indicate relevant differences, it is most likely that pharmacodynamic differences are responsible for different toxicity profiles and response to medication.

### Kinetics of hand–foot syndrome

Figure 1 indicates that Cape-ST is first documented after a median of three treatment cycles. This is in accordance with recent reports stating that grade 2–3 HFS develops after a median of three cycles of therapy (Kang *et al*, 2010; Wolf *et al*, 2010). When the kinetics of Cape-ST was compared with those induced by cetuximab it became apparent that cetuximab-ST developed faster and was documented already after a median of one treatment cycle.

### Predictive value of hand–foot syndrome

So far, there is only limited data regarding the predictive value of HFS as the typical Cape-ST. In the present study, Cape-ST grade 1–3 was not correlated with ORR, however, it predicted a significantly greater DCR (97.9 *vs* 86.1%, *P*=0.038) compared with patients with no Cape-ST. To our knowledge, this is the first publication, which demonstrates the predictive role of Cape-ST for PFS and OS. As this effect was observed in the CAPIRI-C- and the CAPOX-C treatment arm, an interaction with irinotecan or oxaliplatin is unlikely.

### Mechanism of capecitabine-induced hand–foot syndrome

The mechanism of HFS as a main component of Cape-ST remains to be fully understood. Serum levels of 5-FU in patients receiving capecitabine are generally low and therefore do not serve as predictors of HFS (Abushullaih *et al*, 2002). One study examining the levels of TP, a key enzyme in the metabolism of capecitabine to 5-FU, revealed higher TP expression in palmar skin compared with specimens derived from the back of the hand. Accordingly, it may be speculated that activation of capecitabine to the toxic 5-FU occurs to a greater extent in the palmar skin, which then causes HFS (Milano *et al*, 2008). However, it is unknown to what extent palmar toxicity relates to intratumoral activation of capecitabine. A correlation between HFS and treatment efficacy has previously been reported by Twelves *et al* (2008). Patients, receiving capecitabine for adjuvant therapy, who experienced grade 1–3 HFS had a greater 5-year survival rate as compared with patients without HFS (73.8 *vs* 66.3%).

### Limitations

The data provided by this analysis lead to the hypothesis that patients not having any manifestation of HFS might benefit from higher doses of capecitabine. Clearly, the present investigation is limited by its retrospective nature and by the rather small patient number. Furthermore, we cannot completely exclude the possibility that patients showing Cape-ST are also those who are on therapy for longer times and therefore just benefit from longer duration of treatment. As there is much debate about the optimal dosing of capecitabine not only with regard to Cape-ST but also with regard to other toxicities such as diarrhoea, the proposal of a more individualised dosing of capecitabine needs validation by a prospective trial. Also in this context it needs to be kept in mind that regional differences may determine the tolerability profiles of fluoropyrimidines such as capecitabine (Haller *et al*, 2008).

## Conclusion

In the setting of first-line chemotherapy with CAPIRI-C or CAPOX-C, Cape-ST appears to be an early indicator of treatment efficacy. Capecitabine-induced ST is predictive for a longer PFS and OS. The percentage of HFS is associated with higher dosing, so that patients not showing any HFS might be treated with higher doses.

## Figures and Tables

**Table 1:** Capecitabine-specific skin toxicity – frequency of occurrence

	**CAPIRI+cetuximab, (*n*=78)**		**CAPOX+cetuximab, (*n*=71)**			**Both arms (*n*=149)**		
	***n***	**%**	***n***	**%**	***P*-value, Fisher's exact test (two-sided)**	***n***	**%**	
Grade 0^a^	58	74.4	43	60.6	0.81	101	67.8	
Grade 1^a^	9	11.5	7	9.9	0.81	16	10.7	
Grade 2^a^	9	11.5	13	18.3	0.26	22	14.8	
Grade 3^a^	2	2.6	8	11.3	**0.048**	10	6.7	

Abbreviations: CAPIRI=combination of capecitabine and irinotecan; CAPOX=combination of capecitabine with oxaliplatin.

a According to the National Cancer Institute Common Toxicity Criteria of Adverse Events, version 3.0. The bold value indicates statistical significance (*P*<0.05).

**Table 2:** Capecitabine-specific skin toxicity – correlation with response

	**Grade 0, (*n*=101) (67.8%)** a		**Grade 1–3, (*n*=48) (32.2%)** a		***P*-value, Fisher's exact test (two-sided)**
	***n***	**%**	***n***	**%**	
Complete remission (CR)^b^	5	5.0	3	6.3	0.713
Partial remission (PR)^b^	50	49.5	25	52.1	0.861
Stable disease (SD)^b^	32	31.7	19	39.6	0.361
Progressive disease (PD)^b^	14	13.9	1	2.1	0.038
Overall response rate (ORR)^b^	55	54.5	28	58.3	0.725
Disease control rate (DCR)^b^	87	86.1	47	97.9	0.038

a According to the National Cancer Institute Common Toxicity Criteria of Adverse Events, version 3.0.

b According to the RECIST criterion.

**Table 3:** Capecitabine-specific skin toxicity – survival data

	**Grade 0, *n*=101 (78.5%)**	**Grade 1, *n*=16 (10.7%)**	**Grade 2, *n*=22 (14.8%)**	**Grade 3, *n*=10 (6.7%)**	***P*-value logrank** a	**Hazard ratio (95% CI)**
**(A) Capecitabine-related skin toxicities**						
Progression-free survival (PFS) (in months) 95% CI	5.6 (4.8–6.3)	8.5 (5.0–11.9)	8.9 (7.1–10.6)	13.6 (10.4–16.9)	**0.002**	**0.72** **(0.60–0.86)**
Overall survival (OS) (in months) 95% CI	22.4 (17.6–27.2)	30.5 (27.0–33.9)	33.1 (20.8–45.5)	37.5 (24.4–46.0)^b^	0.053	0.74 (0.60–0.92)
				
	**Grade 0, *n*=101 (67.8%)** c		**Grade 1–3, *n*=48 (33.2%)** c	***P*-value logrank**		**Hazard ratio (95% CI)**
**(B) Capecitabine-related skin toxicities, *n*=149**						
Progression-free survival (PFS) (in months) 95% CI	5.6 (4.8–6.3)		9.9 (8.2–11.6)	**<0.001**		**0.51** **(0.36–0.72)**
Overall survival (OS) in months 95% CI	22.4 (17.6–27.2)		32.8 (22.9–42.3)	**0.008**		**0.56** **(0.36–0.86)**
				
	**Grade 0, *n*=58 (74.4%)** c		**Grade 1-3, *n*=20 (25.6%)** c	***P*-value logrank**		**Hazard ratio (95% CI)**
**(C) Capecitabine-related skin toxicities, Arm A (CAIPIRI), *n*=78**						
Progression-free survival (PFS) in months, 95% CI	5.2 (4.5–5.9)		8.5 (5.3–11.7)	**0.011**		**0.52** **(0.31–0.87)**
Overall survival (OS) in months, 95% CI	19.7 (11.9–27.5)		32.0 (25.5–38.5)	0.125		0.63 (0.35–1.14)
				
	**Grade 0, *n*=43 (60.6%)** c		**Grade 1-3, *n*=28 (39.4%)** c	***P*-value logrank**		**Hazard ratio (95% CI)**
**(D) Capecitabine-related skin toxicities, Arm B (CAPOX), *n*=71**						
Progression-free survival (PFS) (in months) 95% CI	6.5 (4.7–8.4)		9.9 (8.3–11.5)	**0.004**		**0.48** **(0.29–0.80)**
Overall survival (OS) (in months) 95% CI	24.0 (11.9–36.1)		37.5 (30.8–46.4)^b^	0.056		0.54 (0.29–1.03)

Abbreviation: CI=confidence interval.

a Logrank for differences of all grades.

b 95% CI of mean as 50% of patients are censored for s.d. of median.

c According to the National Cancer Institute Common Toxicity Criteria of Adverse Events, version 3.0. The bold value in (A) indicates statistically significant (0.002; *P*<0.05); in (B) indicates statistically highly significant (<0.001) and statistically significant (0.008; *P*<0.05); in (C) and (D) indicate statistically significant (0.011 in (C) and 0.004 in (D); *P*<0.05).
